# Design, Synthesis and Cytotoxic Activity of Novel Salicylaldehyde Hydrazones against Leukemia and Breast Cancer

**DOI:** 10.3390/ijms24087352

**Published:** 2023-04-16

**Authors:** Boryana Nikolova-Mladenova, Georgi Momekov, Zvetanka Zhivkova, Irini Doytchinova

**Affiliations:** Faculty of Pharmacy, Medical University of Sofia, 1000 Sofia, Bulgaria; gmomekov@pharmfac.mu-sofia.bg (G.M.); zzhivkova@pharmfac.mu-sofia.bg (Z.Z.); idoytchinova@pharmfac.mu-sofia.bg (I.D.)

**Keywords:** 4-methoxysalicylaldehyde, hydrazones, SAR, cytotoxic activity, HL-60, KE-37, K-562, BV-173, MCF-7, MDA-MB-231, HEK-293

## Abstract

Despite the significant advancements in complex anticancer therapy, the search for new and more efficient specific anticancer agents remains a top priority in the field of drug discovery and development. Here, based on the structure-activity relationships (SARs) of eleven salicylaldehyde hydrazones with anticancer activities, we designed three novel derivatives. The compounds were tested in silico for drug-likeness, synthesized, and evaluated in vitro for anticancer activity and selectivity on four leukemic cell lines (HL-60, KE-37, K-562, and BV-173), one osteosarcomic cell line (SaOS-2), two breast adenocarcinomic cell lines (MCF-7 and MDA-MB-231), and one healthy cell line (HEK-293). The designed compounds were found to have appropriate drug likeness and showed anticancer activities in all cell lines tested; particularly, two of them exhibited remarkable anticancer activity in nanomolar concentrations on the leukemic cell lines HL-60 and K-562 and the breast cancer MCF-7 cells and extraordinary selectivity for the same cancer lines ranging between 164- and 1254-fold. The study also examined the effects of different substituents on the hydrazone scaffold and found that the 4-methoxy salicylic moiety, phenyl, and pyridinyl rings are the most appropriate for anticancer activity and selectivity of this chemical class.

## 1. Introduction

Cancer remains the second leading cause of death worldwide, according to the World Health Organization’s released reports [1]. More than 19 million new cases of cancer and almost 10 million deaths from cancer were estimated worldwide in 2020 [2]. Female breast cancer (2.26 million cases) was the most frequently diagnosed cancer, and new cases of leukemia diagnosed for both sexes are 474.5 thousand [1,2]. Breast cancer is a solid tumor that usually starts in the breast’s ducts or lobules when cells begin to grow out-of-control ductal or lobular carcinomas [3]. The types of cancer also refer to whether cancer has invaded into the surrounding tissue or not: invasive breast cancer (ILS or IDC) and ductal carcinoma in situ (DCIS) [3]. Leukemia is a type of cancer that affects early blood-forming cells and is a significant health concern worldwide. It occurs when the body creates too many abnormal white blood cells and interferes with the bone marrow’s ability to make red blood cells and platelets [3]. Depending on the speed and origin of the abnormal cells, various types of leukemia exist: acute or chronic, lymphocytic or myeloid. Acute lymphocytic leukemia (ALL) and acute myeloid leukemia (AML) start in the bone marrow, but leukemia cells divide rapidly and quickly invade the blood [3,4]. Acute lymphocytic leukemia (ALL) is more common in children, whereas acute myeloid leukemia (AML), chronic myeloid leukemia (CML), and chronic lymphocytic leukemia (CLL) affect mostly adults [4,5].

Treatment of cancer includes a combination of chemotherapy, radiation therapy, targeted therapy, hormone therapy, immunotherapy, and bone marrow transplantation [6]. Many different combinations of anticancer drugs are used to achieve early remission [7,8]. The combination therapeutic approach enhances efficacy compared to the monotherapy approach because it targets important pathways in a manner that is usually additive or synergistic [7]. In addition to therapeutic benefits like reducing tumor growth and metastatic potential, this strategy may also decrease toxicity and lower the risk of drug resistance [7,8,9]. Due to these advantages, combination chemotherapy is now the most used strategy in clinical practice [10].

Over previous years, numerous novel physiologically active compounds have been discovered targeting specific molecular pathways involved in the development and progression of different types of cancer and providing a more targeted approach to treatment. Abemaciclib, palbociclib, and ribociclib are targeted breast cancer drugs that block the activity of two proteins on cancer cells, CDK4 and CDK6. All three drugs are approved to treat advanced or metastatic breast cancer, based on data from large clinical trials showing that they can substantially improve how long patients live without their cancer getting worse [6,11,12,13]. Examples of such drugs for leukemia include tyrosine kinase inhibitors (TKIs), which target abnormal signaling pathways in cancer cells [14,15], and immunotherapeutic agents, which harness the body’s immune system to fight cancer [16]. Several studies have demonstrated the effectiveness of these drugs in treating various types of leukemia. For example, the TKI imatinib has been shown to significantly improve survival rates in patients with chronic myeloid leukemia [17,18], while immunotherapeutic agents, such as CAR-T cell therapy, have shown promising results in the treatment of acute lymphoblastic leukemia [19].

However, there are still several challenges to overcome in the development of drugs against cancer and leukemia. These include drug resistance, toxicity, and the need for personalized treatment approaches. In this context, various hydrazone derivatives have been found to have anticancer effects against breast cancer and leukemia. Hydrazones are derivatives of hydrazine (N_2_H_4_) and contain a C=N bond. These compounds have been found to exhibit a broad range of biological activities, including antifungal, antibacterial, and anticancer properties. Studies have shown that some hydrazones have potent anticancer effects against breast cancer and leukemia cell lines. For example, a series of hydrazones exhibit a prominent effect against MCF-7 breast cancer cells, comparable with the activity of Doxorubicin, a known medication used to treat breast cancer, bladder cancer, lymphoma, and acute lymphocytic leukemia [20,21]. Recently Kaplanek et al. [20] demonstrated that a series of hydrazones exhibited potent cytotoxic effects against human T-lymphoblastic leukemia cells. The researchers found that most of the tested hydrazones exhibit dose-dependent inhibition of both RNA and DNA synthesis, mitosis, and induced apoptosis [22]. Another study by Salum et al. [23] investigated the anticancer properties of a series of hydrazones against acute lymphoblastic leukemia (ALL) cells. Authors found that the hydrazones inhibited the growth of ALL cells and the cell migration. The hydrazones were also found to be less toxic to normal cells, suggesting a potential for selective toxicity against cancer cells [22,23]. While the exact mechanisms of action of hydrazones against cancer cells are not fully understood, studies demonstrate that they may target various cellular processes, including DNA synthesis, cell cycle regulation, and apoptosis. Overall, these results suggest that hydrazones have the potential to be novel anticancer agents. Further research is needed to fully understand their mechanisms of action and potential clinical applications.

During the last few years, we have investigated the anticancer effect of a series hydrazones formed by the condensation of salicylaldehyde derivatives and acyl hydrazides. Salicylaldehyde benzoylhydrazone has been found to decrease DNA synthesis and cell proliferation in a range of cultured human and rodent cells [24,25]. Varieties of substitutions in the salicylaldehyde and hydrazide moieties slightly alter the parent compound but significantly raise the pharmacological properties. The investigations demonstrated that the presence of methoxy group in salicylaldehyde results in derivatives with high antiproliferative activity. 3-methoxysalicylaldehyde-derived hydrazones cause strong cytotoxic effects against the leukemic cell lines, especially those of AML HL-60 cells [26,27]. The presence of a methoxy group in the 5th position in salicylaldehyde hydrazones causes remarkable activity against the solid breast cancer cell line MCF-7 with IC_50_ values of 0.91–3.54 μmol/L [28]. Moreover, these compounds demonstrated selectivity differentiation between malignant and non-tumor cell lines and proved to induce a concentration-dependent increase in the levels of histone-associated DNA fragments, characteristic of apoptosis. These promising results prompted us to replace the methoxy group with other functional groups to check the importance of the substituent. 5-bromosalicylaldehyde-derived hydrazones were highly active against T-cell leukemic cell line SKW-3 and myeloid HL-60 cells, with IC_50_ values of 3.02–3.14 μmol/L, respectively [29,30]. 5-nitrosalicylaldehyde benzoylhydrazones also exhibit high cytotoxic activity in micromolar concentrations against leukemic cell lines HL-60 and BV-173 [31,32].

In continuation of our efforts to discover new hydrazones with pronounced cytotoxic activity, in the present study, we selected a training set of previously synthesized and tested hydrazone derivatives and used them to derive structure-activity relationships (SARs). The conclusions from the SAR analyses guided the design of novel salicylaldehyde hydrazones. Prior to synthesis, the designed compounds were screened in silico for drug likeness. As they showed appropriate drug-like properties, they were synthesized and tested for cytotoxic activity. Two of them showed activities significantly higher than the activities of the compounds from the training set.

## 2. Results

### 2.1. Anticancer Activity of Salicylaldehyde Hydrazones

A set of eleven salicylaldehyde hydrazones previously synthesized and tested for anticancer activity was collected and used as a training set for the SAR analyses (Table 1).

In the present study, we consider the anticancer activity of the compounds on seven cell lines (HL-60, KE-37, K-562, BV-173, SaOS-2, MDA-MB-231, and MCF-7) and express it as IC_50_ values. Some of the activities have been published before [26,27,28,29,30,31,32], the rest were measured for the aim of the present study. The cell lines HL-60, KE-37, K-562, and BV-173 are isolated from patients with different types of leukemia: acute promyelocytic leukemia (HL-60), acute lymphoblastic leukemia (ACC) (KE-37), and chronic myeloid leukemia (K-562 and BV-173), while SaOS-2 cells originate from human osteosarcoma. The cell line MCF-7 is human breast adenocarcinoma expressing estrogen receptor alpha (ER-α) [33], while the cell line MDA-MB-231 represents triple negative breast cancer (TNBC) adenocarcinoma and lacks ER, progesterone receptor (PR), and human epidermal growth factor receptor 2 (HER2) [34].

### 2.2. Structure-Activity Relationship (SAR) Analyses for Salicylaldehyde Hydrazones as Anticancer Agents

The anticancer activities of the compounds from the training set were presented as ligand efficiencies (LE = pIC_50_/number of heavy atoms in molecule) (Table 2) and used to analyze the SAR for each cell line.

Comparing the compounds in terms of average LE, we found that the substituents at position five in R1 increase the efficiency more than the corresponding substituents at position three. Among them, compounds with Br and methoxy substituents are more active than nitro derivatives. Regarding the Ar2 substituents, pyridinyl- and phenyl-substituted hydrazones show higher anticancer efficiency compared to their 4-hydroxyphenyl analogs. These trends persist, with one exception, in the individual cell lines as well. The exception is the ligand efficiency on MCF-7 where the 5-nitro-substituted hydrazones are more efficient than the 5-methoxy analogs. Since the activities were measured on whole cells, no conclusions can be drawn about any specific interaction between a ligand and a target macromolecule.

### 2.3. Design of Novel Hydrazone Derivatives as Anticancer Agents and Drug Likeness Evaluation

Based on the SAR conclusions, we designed a small set of three new hydrazones with the same Ar2 substituents but with a R1 substituent at a different position. We selected to explore position four on the salicylic ring and placed the substituent *OCH*_3_ there (Table 3). Prior to synthesis, the compounds were tested in silico for drug likeness.

The in silico screening for drug likeness included the calculation of the main physicochemical properties (molecular weight Mw, pKa value, fraction of the ionized molecules *f_A_*, *logP,* distribution coefficient at pH 7.4 *logD*_7.4_, polar surface area *PSA*, count of free rotatable bonds *FRB*, hydrogen bond donors *HBD*, hydrogen bond acceptors *HBA*, count of the violations from Lipinski’s Rule of 5 *R5*), the ADME properties (*water solubility*; *GI abs*—gastrointestinal absorption; *oral BA*—oral bioavailability; *BA score*—bioavailability score; *BBB perm*—blood-brain barrier permeability; *CYP inh*—inhibition of CYP enzymes; *P-gp substr*—substrate of P-gp; *drug likeness*; *lead likeness*; *synth access*—synthetic accessibility), and the pharmacokinetic (PK) parameters (fraction of the unbound to plasma proteins molecules *fu*, total clearance *CL*, steady-state volume of distribution *VDss*, and half-life t_1/2_). The calculated values are given in Table 3.

With molecular weights up to 300 g/mol, *logP* around three, five, or six rotatable bonds, and up to three hydrogen-bond donor (*HBD*) groups, the compounds appeared as prospective leads [35]. They behaved as weak acids, existing mainly as neutral molecules at physiological pH 7.4. The PSA is around the limit for BBB permeability [36], distinguishing compound **12** as BBB permeable and compounds **13** and **14**—as non-permeable. The compounds are water soluble with high gastrointestinal absorption and a probability of 55% for the bioavailability to be higher than 10% in rats (BA score) [37]. There is one violation against the criteria for oral bioavailability, concerning the low fraction of Csp3 atoms in molecules. Apart from compound 9, which is predicted to inhibit CYP1A2, the new hydrazones are neither CYP inhibitors nor P-gp substrates. They cover all criteria for drug likeness defined by Lipinski [38], Ghose [39], Veber [40], Egan [41], Muegge [42], and Teague [43]. The synthetic accessibility is around 2.5 corresponding to relatively easy synthesis.

The PK parameters were predicted by previously derived QSPkR models [44,45,46]. The *f_u_* values of 0.2–0.3 suggest high plasma protein binding (97–98%). It is generally accepted that anionic drugs bind primarily with high affinity and capacity to human serum albumin by means of hydrophobic and electrostatic interactions [47]. All compounds have moderate *CL* in the range 0.39–0.46 L/h/kg (according to the classification of Berellini et al.) [48]. The values of *VD_ss_* are relatively low (0.20–0.21 L/kg), suggesting distribution mainly in extravascular body water. The low *VD_ss_* and moderate *CL* determine short *t*_1/2_ of the new hydrazine derivatives–between 0.30 and 0.36 h.

### 2.4. Synthesis and Characterization of the Newly Designed 4-Methoxysalicylaldehyde Hydrazones

**Scheme 1:**
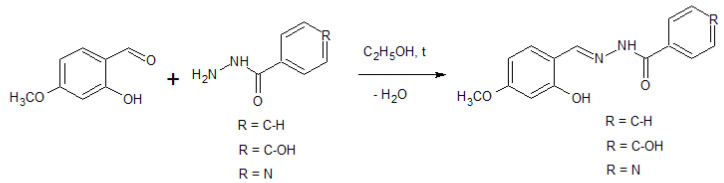
Synthesis of the 4-methoxysalicylaldehyde-based hydrazones **12** (X = C-H), **13** (X = C-OH), and **14** (X = N)–H_2_O.

The 4-methoxysalicylaldehyde-based hydrazones were synthesized by the reaction of Schiff base condensation in ethanol, as previously described [26,27,28,29] according to Scheme 1. The hydrazones were obtained in high yields and their structures were identified using a variety of analytical and spectroscopic techniques. The melting points and the elemental analysis of the hydrazones, as well as HR ESI–MS, IR, ^1^H NMR, and ^13^C NMR data are given in the Experimental section.

The elemental analysis suggests the hydrazones’ molecular formulae. Mass spectra and thermal investigations of the obtained compounds confirmed the composition. The hydrazones’ HR ESI–MS spectra revealed protonated molecular ions [M + H]^+^, which correspond to the suggested molecular formulae. TGA and DTA data were used to determine the H_2_O molecule content. Compounds **12** and **13** are anhydrous, whereas compound **14** includes one H_2_O molecule and loses it at 90–150 °C.

The structures of the newly synthesized hydrazones were found through IR and NMR spectroscopy. The IR spectra of the hydrazones show an intensive band around 1602–1609 cm^−1^ assigned to the azomethine group C=N, which proves the condensation between the aldehyde group of 4-methoxysalicylaldehyde and the amino group of hydrazides in the formation of Schiff base. The medium intensity peak around 3350–3440 cm^−1^ and the weak broadband at 3213–3228 cm^−1^ were assigned to the phenolic hydroxyl group and NH group, respectively. The intensive characteristic bands at 1640–1662 cm^−1^ in the spectra of the compounds were assigned to the frequency vibration of the carbonyl group C=O and suggest the existence of the hydrazones in keto form in solid state. Another important band at 1578–1600 cm^−1^ was attributed to n(C-NH).

The hydrazones were further studied by their ^1^H NMR and ^13^C NMR spectra in deuterated dimethyl sulfoxide (DMSO). The ^1^H NMR spectra revealed the presence of aromatic protons in the region δ 6.50–7.81. The protons of the methoxy group appeared as a singlet at δ 3.72–3.75. Signals for the protons of the characteristic for hydrazone azomethine group HC=N- were observed at δ 8.56, 8.51, and 8.58, respectively, for compounds **12**, **13,** and **14**. The broad singlets around δ 11.78–12.18 were assigned to the protons of the hydroxyl group from the aldehyde ring. The ^13^C NMR spectra demonstrated the signals corresponding to the carbon atoms of azomethine group at δ 148.87, 148.10, and 150.33, respectively, for **12**, **13**, and **14**. The peaks at δ 162.59, 161.86, and 161.07 were assigned to the C=O group. The ^1^H NMR and ^13^C NMR data in deuterated DMSO give additional information for the new hydrazones.

### 2.5. Anticancer Activities of the Newly Designed 4-Methoxysalicylaldehyde Hydrazones

The newly synthesized derivatives were tested for cytotoxic activity on the same cell lines. Throughout the screening study, the data about the 4-methoxy hydrazones were compared with the clinically used antineoplastic drug, Melphalan (2-amino-3-[4-bis(2-chloroethyl)amino]phenylpropanoic acid). The tested compounds inhibited the proliferation of cells in a concentration-dependent manner, which enabled the construction of concentration-response curves. The experimental values for IC_50_ are given in Table 4.

As demonstrated from the results obtained, all cell lines were significantly more sensitive to 4-methoxy hydrazone derivatives than to the reference anticancer drug, Melphalan. The three 4-methoxy hydrazones showed very high cell growth inhibition in human leukemic cell lines. The compounds most strongly decreased the proliferation of cells from the chronic myeloid leukemia line K-562. The concentration-effect curves gave IC_50_ values of 0.03 μM and 0.05 μM for hydrazones **12** and **14**, respectively. These were much lower (more than 900-fold) than those obtained for typical cytostatic Melphalan. The results on the acute leukemia cell line HL-60 were similar. The experimental IC_50_ values for compounds **12** and **14** were 0.04 μM and 0.06 μM. The cytotoxic activity of the 4-methoxy-derived hydrazones in chronic myeloid leukemia BV-173 and acute lymphoblastic leukemia KE-37 cells revealed that hydrazones produced comparable cytotoxic effects, with compound **13** being less active according to the IC_50_ values (Table 4). On the SaOS-2 tumor line, the investigated compounds exhibited concentration-dependent cytotoxic activity and a significant reduction in the number of vital cells was observed even at the lowest applied concentration. The determined IC_50_ values were lower than those of Melphalan.

The solid-tumor cell lines MDA-MB-231 and MCF-7 were also more sensitive to 4-methoxy hydrazone derivatives than the reference anticancer drug. The breast cancer MCF-7 cell line was the most sensitive. The compounds **12** and **14** exhibited greater cytotoxicity on MCF-7 cells, with an IC_50_ value of 0.23 μM. They inhibited cell growth by 50% at approximately 500-fold lower concentrations than Melphalan. All three 4-methoxy-derived hydrazones inhibited the growth of the MDA-MB-231 solid tumor breast cancer cell line. The compounds effectively reduced the percentage of viable MDA-MB-231 cells and displayed comparable cytotoxic effects, with IC_50_ values ranging from 1.22–2.90 μM.

### 2.6. Selectivity of the Newly Designed 4-Methoxysalicylaldehyde Hydrazones

To assess the selectivity of the newly synthesized derivatives, they were tested on the normal human embryonic kidney HEK-293 cell line. The derived IC_50_ values were used to calculate the selectivity index according to the formula *SI* = IC_50_ (normal cells)/IC_50_ (cancer cells). The *SI* values are given in Table 4. They range from 2 (for compound **13** on SaOS-2) to 1254 (for compound **12** on K-562). The most selective is compound **12**, with an average *SI* of 350, followed by compound **14**, with average *SI* of 258. Compound **13** was the least selective, with an average *SI* of 14. All three new hydrazones were more selective than the positive control, Melphalan.

## 3. Discussion

The findings presented here are a continuation of our previous research on salicylaldehyde hydrazones with anticancer activity [26,27,28,29,30,31,32]. A set of eleven compounds synthesized and tested before was used as a training set and SAR analyses were performed for cytotoxic activity on HL-60, KE-37, K-562, BV-173, SaOS-2, and MCF-7 cell lines. Based on the SAR conclusions, three novel derivatives were designed, their drug likeness was confirmed by in silico calculations, and then they were synthesized and tested. All three compounds showed anticancer activities in the lower micromolar range which were higher than the activities of the compounds from the training set and the positive control, Melphalan.

The designed 4-metoxysalicylic hydrazones are weak acids with pK_a_ values in the range of 7.67–8.08, existing at physiological pH 7.4 as 65–83% non-ionized molecules. They meet the requirements for drug and lead likeness, have small *VD_ss_,* moderate *CL*, and short half-lives. In terms of anticancer activity, compounds **12** and **14** are more active than compound **13** in all cell lines. The highest activity is demonstrated on the leukemic cell lines HL-60 and K-562, where the measured IC_50_ values are below 60 nM. Similar extreme activity is observed on the breast cancer cell line MCF-7, with IC_50_ of 230 nM for compounds **12** and **14**. On the leukemic cell lines, both compounds are equipotent, on the triple negative breast cancer cell line MDA-MB-231, compound **14** is twice more active than compound **12** (1.22 µM vs. 2.31 µM).

The selectivity for cancer cells of the tested compounds ranges in quite wide limits, from 2 to 1254, expressed as *SI*. In terms of selectivity, compound **12** showed the best performance, being 1254-, 941- and 164-fold more toxic on K-562, HL-60, and MCF-7, respectively, than on normal cells. Close to it, compound **14** is the next most selective with *SI*s equal to 821, 685, and 179 on the same cell lines. The least selective compound was hydrazone **13**.

The close anticancer activities and selectivities of compounds **12** and **14** on leukemic cells indicate that benzoyl and isonicotinoyl substituents at the Ar2 position of the hydrazone scaffold are bioisosteric. However, incorporation of a hydroxy group into the benzoyl ring (compound **13**) reduced cytotoxicity between 3- (BV-173) and 292-fold (K-562). A moderate decrease is observed on sarcomic cells (15.5-fold reduction) and on breast cancer cells (1.25-fold reduction for MDA-MB-231 and 6-fold reduction for MCF-7).

Regarding the substituent at position R1, the 4-methoxysalicilic moiety was found to be the most appropriate for anticancer activity compared to 3-methoxy, 5-bromo, and 5-nitro-substituted salicylic hydrazones.

Overall, this study provides valuable insights into the design and development of new hydrazone-based compounds with potential anticancer activity. The results of this study clearly guide further research into hydrazone derivatives as potential and selective anticancer agents and contribute to the development of more effective cancer treatments.

## 4. Materials and Methods

### 4.1. Datasets

The training set of salicylaldehyde hydrazones contained 11 compounds synthesized and tested before in our Lab [26,27,28,29,30,31,32]. Their anticancer activities were measured and expressed as IC_50_ values on the following cell lines: HL-60, KE-37, K-562, BV-173, SaOS-2, MDA-MB-231, and MCF-7. For the purposes of SAR analysis, the IC_50_ values were converted in p-units (−logIC_50_) and ligand efficiencies (LEs) were calculated according to the formula: *LE* = pIC_50_/*n*, where *n* is the count of non-hydrogen atoms in the molecule.

### 4.2. ADME and PK Properties

The physicochemical properties of the designed compounds were calculated by the ACD/LogD tool v. 9.08 (Advanced Chemistry Development Inc., Toronto, ON, Canada). The ADME properties were calculated by the SwissADME tool [36]. The PK parameters were calculated by previously derived QSPkR models [44,45,46] for the fraction of molecules unbound to plasma proteins, *f_u_*; unbound clearance, *CL_u_*; and steady state volume of distribution, *VD_ss_*. The datasets consisted of 132 acidic drugs, extracted from Obach’s database–the largest and best curated source of data for the key pharmacokinetic parameters after iv administration [49]. Chemical structures of the compounds were encoded by 178 molecular descriptors calculated by ACD/logD version 9.08 (Advanced Chemistry Development Inc., Toronto, ON, Canada) and MDL QSAR version 2.2 (MDL Information Systems Inc, San Leandro, CA, USA). Genetic algorithm and stepwise multiple linear regression were applied for variable selection and model derivation. The QSPkRs were evaluated by internal and external validation procedures.

### 4.3. Preparation and Characterization

All used compounds were of analytical reagent grade. 4-methoxysalicylaldehyde, 4-hydroxybenzhydrazide, isonicotinic hydrazide, and 96% ethanol were purchased from Merck (Darmstadt, Germany) and used without further purification. The carbon, nitrogen, and hydrogen contents of the compounds were determined by elemental analyses on a Euro EA 3000-Single, EuroVector SpA (Milan, Italy). The melting points were determined using a Buchi 535 apparatus (Flawil, Switzerland). The IR spectra in the range of 4000–400 cm^−1^ were recorded using KBr pallets on a Bruker Tensor 27 spectrophotometer (Ettlingen, Germany). The ^1^H and ^13^C NMR spectra were recorded on a Bruker Avance DRX 250 spectrophotometer (Rheinstetten, Germany) in DMSO-*d_6_* as a solvent, using tetramethylsilane (TMS) as an internal standard. Chemical shifts (δ) were reported in parts per million (ppm); *J* values were given in Hz. Splitting patterns were indicated by the symbols: s (singlet), d (doublet), t (triplet), and m (multiplet).

#### Synthesis of the Hydrazones

A solution of 4-methoxysalicylaldehyde (0.01 mol) in 96% ethanol (10 mL) was added to the solutions of the corresponding benzhydrazides (0.01 mol) in 50% aqueous ethanol (40 mL) and immediately precipitates formed. An extra 96% ethanol was added, and the mixtures were stirred and heated for 20 min at 60 °C. The obtained solutions were allowed to cool and stand at room temperature for 24 h. During this time, crystals of the products were obtained, then filtered off. The solid hydrazones were dried for 2 days in a vacuum desiccator.

4-methoxysalicylaldehyde benzoylhydrazone, compound **12**.

Yield: 84%; mp: 182–183 °C; Color: Pale yellow; IR (ν cm^−1^): 3440 (OH), 3228 (NH), 1640 (C=O), 1602 (C=N), 1578 (C-NH). ^1^H NMR (250 MHz, DMSO-*d_6_*) δ ppm: 3.78 (s, 3H, OCH_3_), 6.53 (d, 2H, *J* = 8.75 Hz, ArH_aldehyde_), 7.42 (d, 1H, *J* = 8.75 Hz, ArH_aldehyde_), 7.57 (m, 3H, ArH_hydrazide_), 7.93 (d, 2H, *J* = 8.25 Hz, ArH_hydrazide_), 8.56 (s, 1H, CH=N), 11.65 (s, 1H, NH), 11.99 (s, 1H, OH). ^13^C NMR (250 MHz, DMSO-*d_6_*) δ ppm: 55.28 (OCH_3_), 112.28, 117.24, 118.20, 118.93, 127.62, 128.51, 131.92, 132.87, 148.87 (CH=N), 151.47, 152.12, 162.59 (C=O). HR ESI–MS *m*/*z*: 271.10734 [M + H]^+^. Calculated for C_15_H_14_N_2_O_3_: C, 66.66; H, 5.22; N, 10.36. Found: C, 67.02; H, 5.07; N, 10.44.

4-methoxysalicylaldehyde-4-hydroxybenzoylhydrazone, compound **13**.

Yield: 80%; mp: 216–217 °C; Color: Pale yellow; IR (ν cm^−1^): 3400 (OH), 3300 (OH), 3213 (NH), 1662 (C=O), 1608 (C=N), 1589 (C-NH). ^1^H NMR (250 MHz, DMSO-*d_6_*) δ ppm: 3.77 (s, 3H, OCH_3_), 6.50 (s, 1H, ArH_aldehyde_), 6.51 (d, 1H, *J* = 9 Hz, ArH_aldehyde_), 7.38 (d, 1H, *J* = 9 Hz, ArH_aldehyde_), 6.87 (d, 2H, *J* = 8.75 Hz, ArH_hydrazide_), 7.81 (d, 2H, *J* = 8.5 Hz, ArH_hydrazide_), 8.51 (s, 1H, CH=N), 10.13 (s, 1H, NH), 10.13 (s, 1H, OH_hydrazide_), 11.78 (s, 1H, OH_aldehyde_); ^13^C NMR (250 MHz, DMSO-*d_6_*) δ ppm: 55.26 (OCH_3_), 112.52, 115.07, 117.18, 117.86, 118.95, 123.27, 129.72, 148.10 (CH=N), 151.39, 152.08, 160.84, 161.86 (C=O). HR ESI–MS *m*/*z*: 287.10223 [M + H]^+^. Calculated for C_15_H_14_N_2_O_4_: C, 62.93; H, 4.93; N, 9.79. Found: C, 62.98; H, 5.07; N, 9.68.

4-methoxysalicylaldehyde isonicotinoylhydrazone, compound **14**.

Yield: 97%; mp: 232–233 °C; Color: Bright yellow; IR (ν cm^−1^): 3350 (OH), 3190 (NH), 1658 (C=O), 1609 (C=N), 1600 (C-NH). ^1^H NMR (250 MHz, DMSO-*d_6_*) δ ppm: 3.78 (s, 3H, OCH_3_), 6.51 (s, 1H, ArH_aldehyde_), 6.53 (d, 1H, *J* = 8.75 Hz, ArH_aldehyde_), 7.47 (d, 1H, *J* = 8.75 Hz, ArH_aldehyde_), 7.83 (d, 2H, *J* = 6 Hz, ArH_hydrazide_), 8.58 (s, 1H, CH=N), 8.78 (d, 2H, *J* = 6 Hz, ArH_hydrazide_), 11.43 (s, 1H, NH), 12.18 (s, 1H, OH). ^13^C NMR (250 MHz, DMSO-*d_6_*) δ ppm: 55.30 (OCH_3_), 111.77, 117.31, 118.64, 118.93, 121.49, 140.04, 150.33 (CH=N), 150.33, 151.50, 152.16, 161.07 (C=O). HR ESI–MS m/z: 272.10267 [M + H]^+^. Calculated for C_14_H_13_N_3_O_3_.H_2_O: C, 58.13; H, 5.23; N, 14.53. Found: C, 58.47; H, 5.27; N, 14.49.

The IR and NMR spectra of the hydrazones **12**–**14** are presented in Supplementary, Figures S1–S9.

### 4.4. Cytotoxic Activity

#### 4.4.1. Cell Lines and Culture Conditions

The research included four leukemic and three cancer human cell lines: HL-60 (acute myeloid leukemia), SKW-3 (KE-37 derivative) (T-cell leukemia), BV-173 and K-562 (chronic myeloid leukemia), MDA-MB-231 (ER-negative breast carcinoma), MCF-7 (ER-positive breast adenocarcinoma), and SaOS-2 (osteogenic sarcoma). The cells were provided from the German Collection of Microorganisms and Cell Cultures (DSMZ, Braunschweig, Germany). The solid tumor cell lines (MDA-MB-231, MCF-7, and SaOS-2) were incubated in 90% RPMI-1640 supplemented with 10% fetal bovine serum (FBS), non-essential amino acids, 1 mM sodium pyruvate, and 10 mg/mL human insulin as monolayer adherent cultures. The remaining cell lines were cultivated as a suspension culture in cell culture flasks at 37 °C in an incubator “BB 16-Function Line” Heraeus (Kendro, Hanau, Germany) with humidified atmosphere and 5% CO_2_ under standard conditions–RPMI-1640 liquid media supplemented with 10% FBS and 2 mM L-glutamine. The cells were kept in log phase by supplementing with fresh media after removing cell suspension aliquots two or three times each week.

#### 4.4.2. Cytotoxicity Assessment (MTT-Dye Reduction Assay)

The cytotoxic activity of the examined substances was determined using Mossman’s [50] MTT [3-(4,5-dimethylthiazol-2-yl)-2,5-diphenyltetrazolium bromide] dye reduction assay. The approach is based on the reduction of the yellow tetrazolium salt MTT to a violet formazan in live cells via mitochondrial succinate dehydrogenase. In brief, exponentially developing cells were seeded at a density of 1 × 10^5^ cells per ml in 96-well flat-bottomed microplates (100 μL/well), and after 24 h of incubation at 37 °C, they were treated to varied doses of the tested substances for 72 h. At least eight wells were used for each concentration. Following the incubation with the test compounds, aliquots of 10 μL MTT solution (10 mg/mL in PBS) were applied to each well. The microplates were then incubated at 37 °C for 4 h before the MTT-formazan crystals were dissolved with 100 μL/well 5% HCOOH in 2-propanol. The MTT-formazan absorbance was measured at 580 nm using a microprocessor-controlled microplate reader (Labexim LMR-1).

#### 4.4.3. Data Processing and Statistics

The cell survival data were standardized as a percentage of the untreated control (set as 100% viability). The biological data were processed statistically using Student’s t-test, where values were considered statistically significant at *p* ≤ 0.05. The corresponding IC_50_ values were obtained to allow quantitative merit for the assessment of the relative potencies of the compounds under investigation. Each data point represents the arithmetic mean ± standard deviation (sd) of at least eight independent experiments. The IC_50_ values were obtained by non-linear regression analysis from the concentration-response curves and represent the concentrations of the tested compounds causing a 50% decrease in cell survival.

## Figures and Tables

**Table 1:** IC_50_ values ± standard deviations of the compounds from the training set used in the SAR analyses. The anticancer activities of the compounds were measured on four leukemia cell lines (HL-60, KE-37, K-562, and BV-173), one osteosarcoma cell line (SaOS-2), one breast ER+ adenocarcinoma cell line (MCF-7), and one TNBC cell line (MDA-MB-231). Melphalan was used as a positive control.

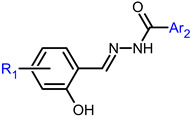									
ID	R1	Ar2	HL-60	KE-37	K-562	BV-173	SaOS-2	MDA-MB-231	MCF-7
1	3-methoxy-	phenyl	11.30 ± 2.6	9.80 ± 3.1	27.00 ± 2.7	20.8 ± 4.2	25.50 ± 6.2	25.30± 2.7	22.50 ± 5.4
2	3-methoxy-	4-hydroxyphenyl	9.20 ± 2.4	7.60 ± 2.1	14.30 ± 2.9	7.8 ± 2.0	19.20± 3.1	24.20 ± 3.8	19.70 ± 2.2
3	3-methoxy-	pyridine-4-yl	2.70 ± 0.9	3.30 ± 1.2	8.70 ± 2.0	3.7 ± 1.1	11.90 ± 3.2	16.40 ± 2.0	11.70 ± 2.4
4	5-bromo-	4-hydroxyphenyl	3.14 ± 1.1	3.00 ± 0.5	2.83 ± 0.3	2.0 2± 0.9	49.96 ± 5.7	20.10 ± 3.9	8.88 ± 2.7
5	5-bromo-	pyridine-4-yl	4.13 ± 1.2	2.53 ± 1.0	0.14 ± 0.09	0.63 ± 0.2	146.20 ± 8.9	38.40 ± 2.0	0.26 ± 0.03
6	5-nitro-	phenyl	5.64 ± 1.1	6.38 ± 0.2	7.94 ± 2.3	2.39 ± 0.3	41.88 ± 5.1	2.80 ± 2.7	0.25 ± 0.03
7	5-nitro-	4-hydroxyphenyl	14.46 ± 2.1	19.90 ± 2.2	>200	7.62 ± 1.0	198.60 ± 12.0	>200	4.95 ± 0.06
8	5-nitro-	pyridine-4-yl	14.97 ± 2.4	16.98 ± 3.1	18.98 ± 2.7	10.19 ± 1.1	35.69 ± 4.3	72.30 ± 5.6	0.18 ± 0.03
9	5-methoxy-	phenyl	5.29 ± 1.2	3.38 ± 1.0	na ^1^	3.15 ± 0.8	na	4.46 ± 1.1	0.91 ± 0.3
10	5-methoxy-	4-hydroxyphenyl	13.80 ± 3.1	14.80 ± 2.5	na	3.86 ± 0.9	na	12.07 ± 1.9	3.54 ± 0.5
11	5-methoxy-	pyridine-4-yl	6.68 ± 1.4	10.86 ± 2.2	na	2.95 ± 0.8	na	5.58 ± 1.3	3.00 ± 0.6
Melphalan			18.50 ± 2.1	21.40 ± 3.9	28.2 ± 7.1	31.30 ± 3.9	31.40 ± 5.4	42.20 ± 3.7	33.70 ± 4.2

^1^ na—not available.

**Table 2:** Ligand efficiencies of the hydrazone derivatives from the training set.

Compound ID	R1	Ar2	Ligand Efficiency (LE)							
			HL-60	KE-37	K-562	BV-173	SaOS-2	MDA-MB-231	MCF-7	Average
1	3-methoxy-	phenyl	0.247	0.250	0.228	0.234	0.230	0.230	0.232	0.236
2	3-methoxy-	4-hydroxyphenyl	0.240	0.244	0.231	0.243	0.225	0.220	0.224	0.232
3	3-methoxy-	pyridine-4-yl	0.278	0.274	0.253	0.272	0.246	0.239	0.247	0.258
4	5-bromo-	4-hydroxybenzoyl	0.275	0.276	0.277	0.285	0.215	0.235	0.253	0.259
5	5-bromo-	4-hydroxyphenyl	0.283	0.295	0.360	0.326	0.202	0.232	0.346	0.292
6	5-nitro-	pyridine-4-yl	0.250	0.247	0.243	0.268	0.208	0.264	0.315	0.256
7	5-nitro-	4-hydroxyphenyl	0.220	0.214	na ^1^	0.233	0.168	na	0.241	0.215
8	5-nitro-	pyridine-4-yl	0.230	0.227	0.225	0.238	0.212	0.197	0.321	0.235
9	5-methoxy-	phenyl	0.264	0.274	na	0.275	na	0.268	0.302	0.276
10	5-methoxy-	4-hydroxyphenyl	0.231	0.230	na	0.258	na	0.234	0.260	0.243
11	5-methoxy-	pyridine-4-yl	0.259	0.248	na	0.277	na	0.263	0.276	0.264

^1^ na—not available.

**Table 3:** Physicochemical and ADME properties and pharmacokinetic parameters of 4-metoxysalicylic hydrazones.

Parameter	Compound 12	Compound 13	Compound 14
R1	4-methoxy-	4-methoxy-	4-methoxy-
Ar2	phenyl	4-hydroxyphenyl	pyridine-4-yl
*Mw* g/mol	270.28	286.28	271.27
*pKa*	7.8	8.08	7.67
*f_A_*	0.28	0.17	0.35
*logP*	3.69	3.07	2.41
*logD*_7.4_	3.58	2.85	2.28
*PSA* Å	70.92	91.15	83.81
*FRB*	5	6	5
*HBD*	2	3	2
*HBA*	5	6	6
*R5*	Yes	Yes	Yes
*water solubility*	Moderately soluble	Soluble	Soluble
*GI absorption*	High	High	High
*oral BA*	INSATU	INSATU	INSATU
*BA score*	0.55	0.55	0.55
*BBB permeability*	Yes	No	No
*CYP inhibition*	1A2	No	No
*P-gp substrate*	No	No	No
*Drug likeness*	Yes	Yes	Yes
*Lead likeness*	Yes	Yes	Yes
*Synth access*	2.46	2.53	2.43
*fu*	0.02	0.02	0.03
*CL* L/h/kg	0.39	0.42	0.46
*VDss* L/kg	0.21	0.20	0.21
*t*_1/2_ h	0.36	0.34	0.30

**Table 4:** Experimental IC_50_ values in μM and selectivity indices (*SI*) for cytotoxic activities of 4-metoxysalicylic hydrazones after 72 h treatment.

Parameter	Cell Line	Compound 12	Compound 13	Compound 14	Melphalan
IC_50_ μM exp	HL-60	0.04 ± 0.005	8.90 ± 2.47	0.06 ± 0.004	18.50 ± 2.1
	KE-37	1.99 ± 0.17	10.83 ± 2.81	2.26 ± 0.73	21.40 ± 3.9
	K-562	0.03 ± 0.002	8.75 ± 1.29	0.05 ± 0.008	28.20 ± 7.1
	BV-173	1.21 ± 0.11	3.88 ± 1.16	0.97 ± 0.12	31.30 ± 3.9
	SaOS-2	1.68 ± 0.15	26.01 ± 3.65	1.66 ± 0.24	31.40 ± 5.4
	MDA-MB-231	2.31 ± 0.25	2.90 ± 0.32	1.22 ± 0.19	42.20 ± 3.7
	MCF-7	0.23 ± 0.02	1.38 ± 0.08	0.23 ± 0.04	33.70 ± 4.2
	HEK-293	37.63 ± 3.41	56.67 ± 4.35	41.07 ± 2.05	24.81 ± 2.90
*SI*	HL-60	941	6	685	1
	KE-37	19	5	18	1
	K-562	1254	6	821	1
	BV-173	31	15	42	1
	SaOS-2	22	2	25	1
	MDA-MB-231	16	20	34	2
	MCF-7	164	41	179	1

## Data Availability

Not applicable.

## Supplementary Materials

The following supporting information can be downloaded at: https://www.mdpi.com/article/10.3390/ijms24087352/s1.
