# Supplementary material for: Design, Synthesis and Cytotoxic Activity of Novel Salicylaldehyde Hydrazones against Leukemia and Breast Cancer
Source: Int J Mol Sci. 2023 Apr 16;24(8):7352. doi: 10.3390/ijms24087352 (PMC10138506; doi:10.3390/ijms24087352)
Supplement: Supplementary file 1 [file ijms-24-07352-s001.zip › ijms-2327006-supplementary.pdf]

Supplementary Material

Design, Synthesis and Cytotoxic Activity of Novel Salicylaldehyde Hydrazones against Leukemia and Breast Cancer

Boryana Nikolova-Mladenova [1*], Georgi Momekov [2], Zvetanka Zhivkova [1] and Irini Doytchinova [1]

[1] Department of Chemistry, Faculty of Pharmacy, Medical University of Sofia, 2 Dunav str., Sofia, Bulgaria; boriananik@pharmfac.mu-sofia.bg; zzhivkova@pharmfac.mu-sofia.bg; idoytchinova@pharmfac.mu-sofia.bg

[2] Department of Pharmacology, Pharmacotherapy and Toxicology, Faculty of Pharmacy, Medical University of Sofia, 2 Dunav str., Sofia, Bulgaria; gmomekov@pharmfac.mu-sofia.bg

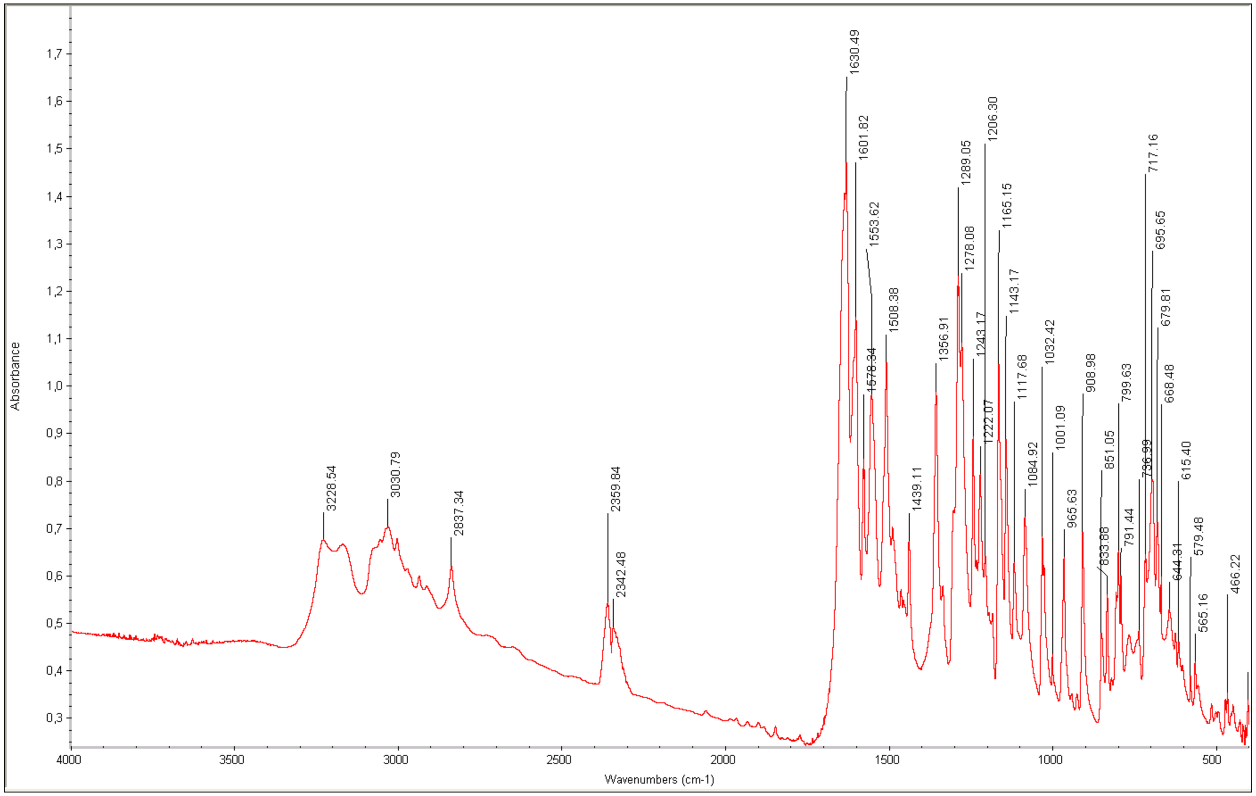

Figure S1. IR spectra of the compound **12**

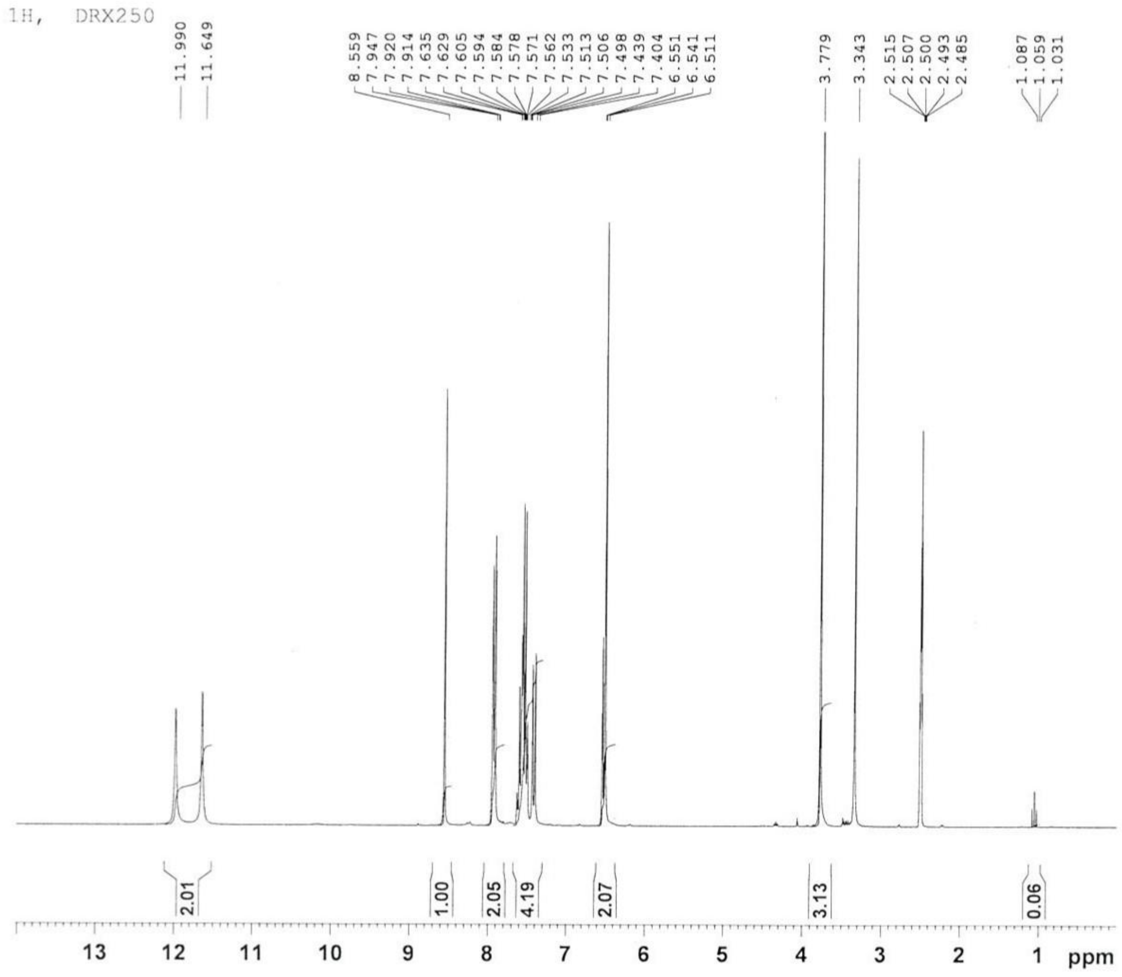

Figure S2. ^{1}H NMR spectra of the compound **12**

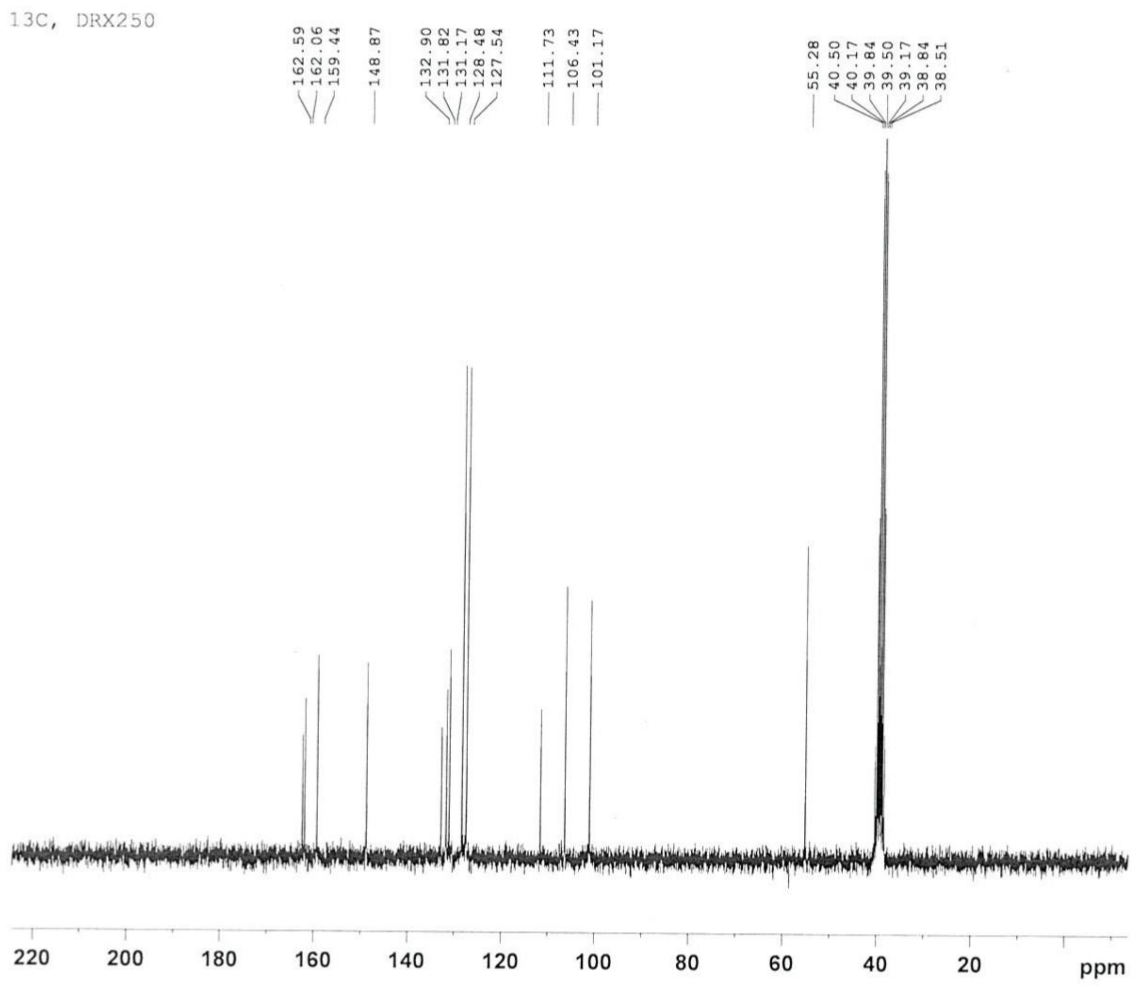

Figure S3. ^{13}C NMR spectra of the compound **12**

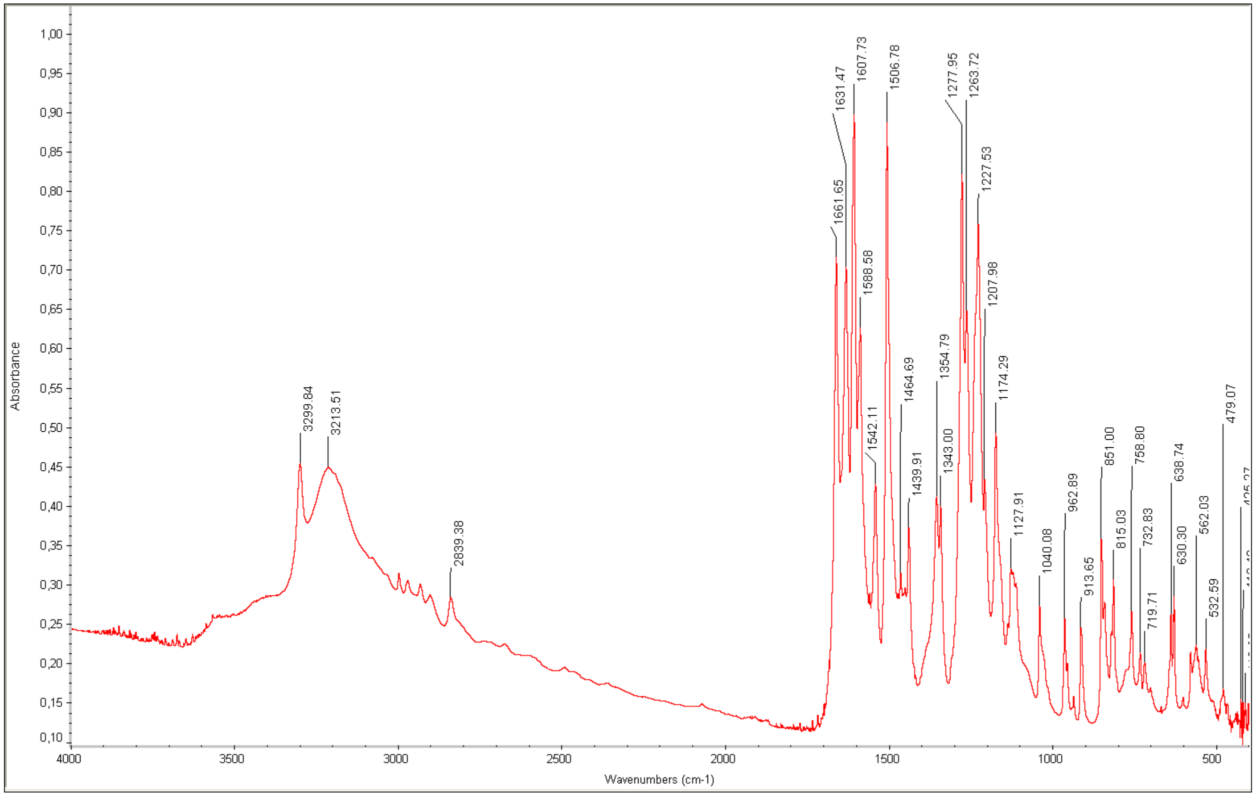

Figure S4. IR spectra of the compound **13**

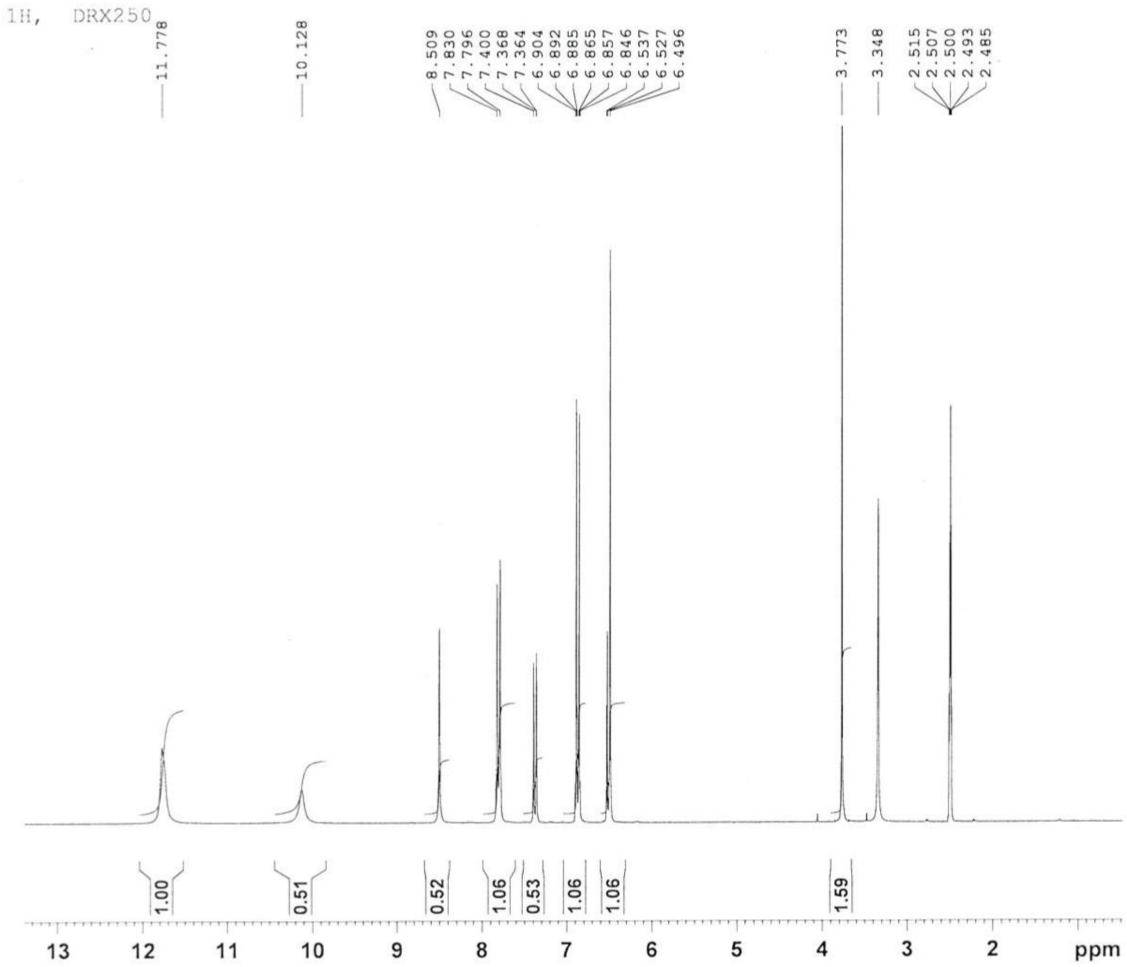

Figure S5. ^{1}H NMR spectra of the compound **13**

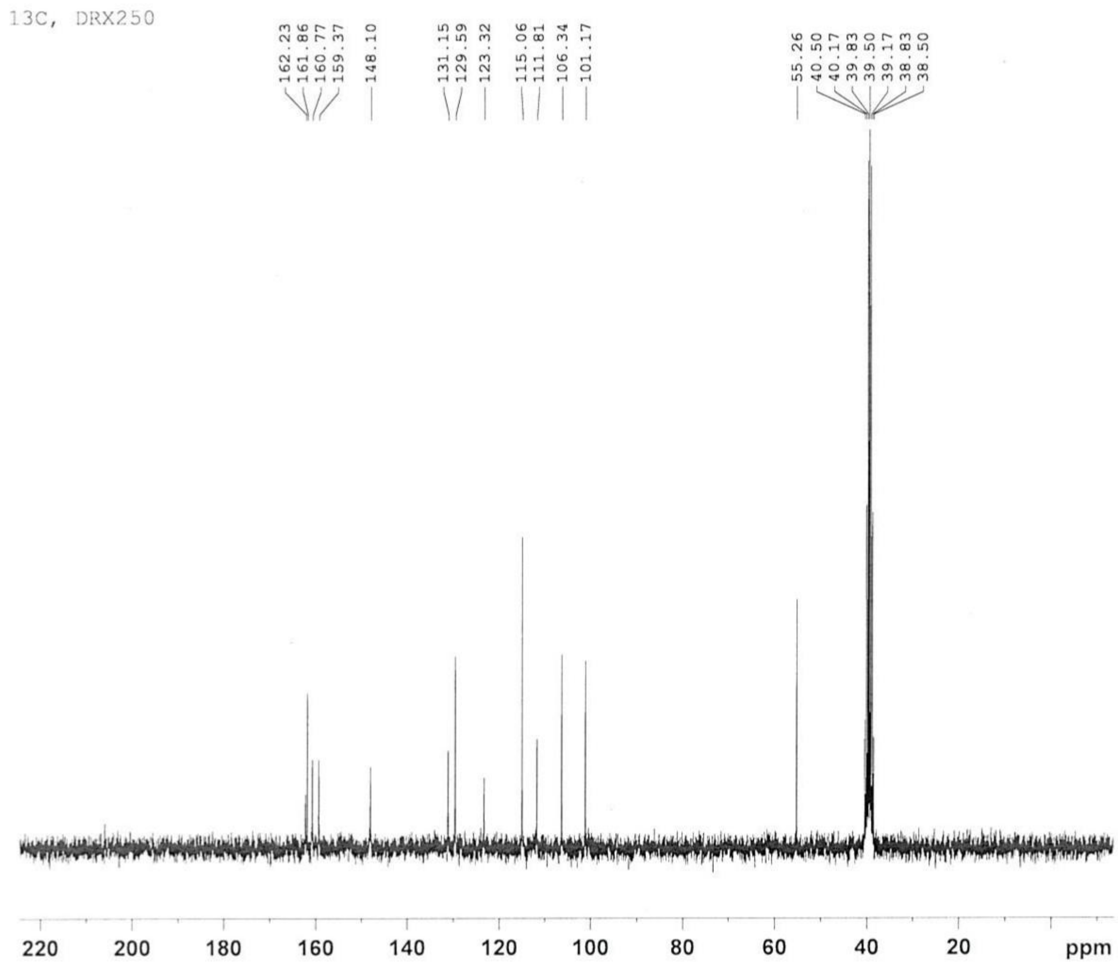

Figure S6. ^{13}C NMR spectra of the compound **13**

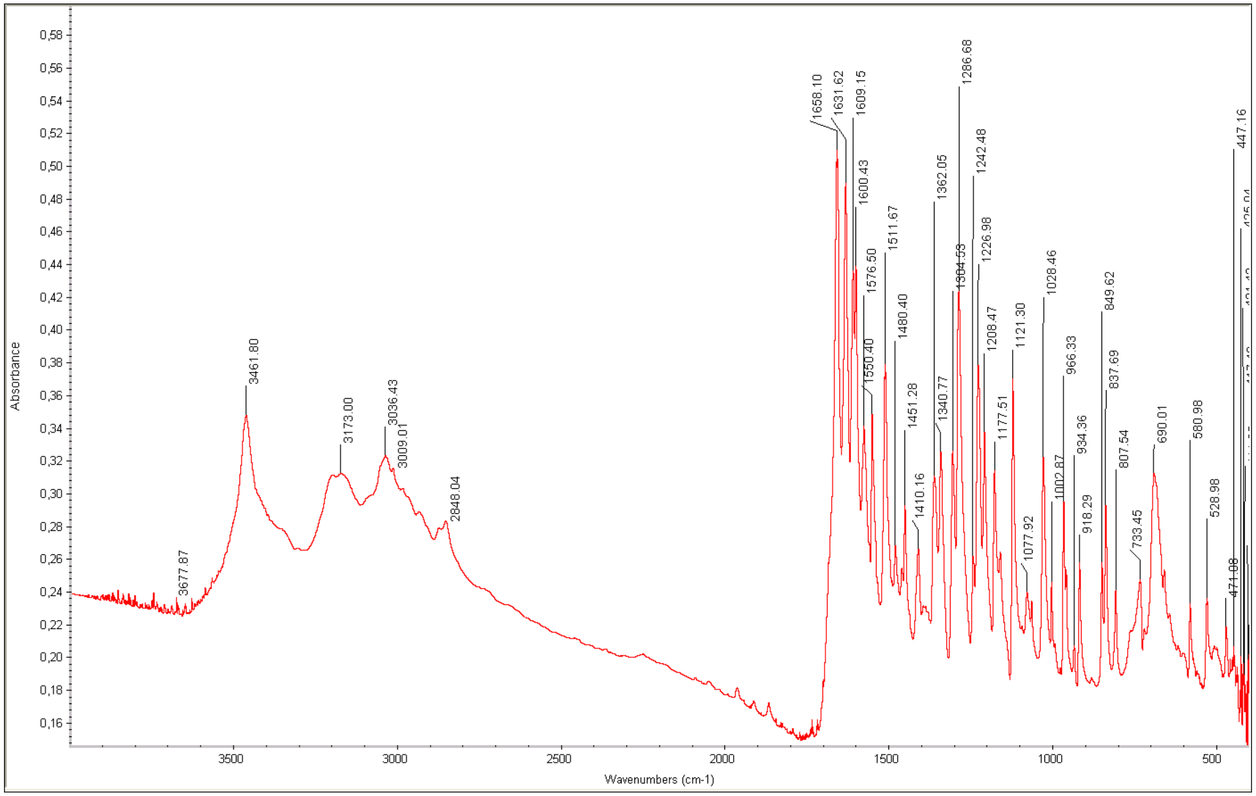

Figure S7. IR spectra of the compound **14**

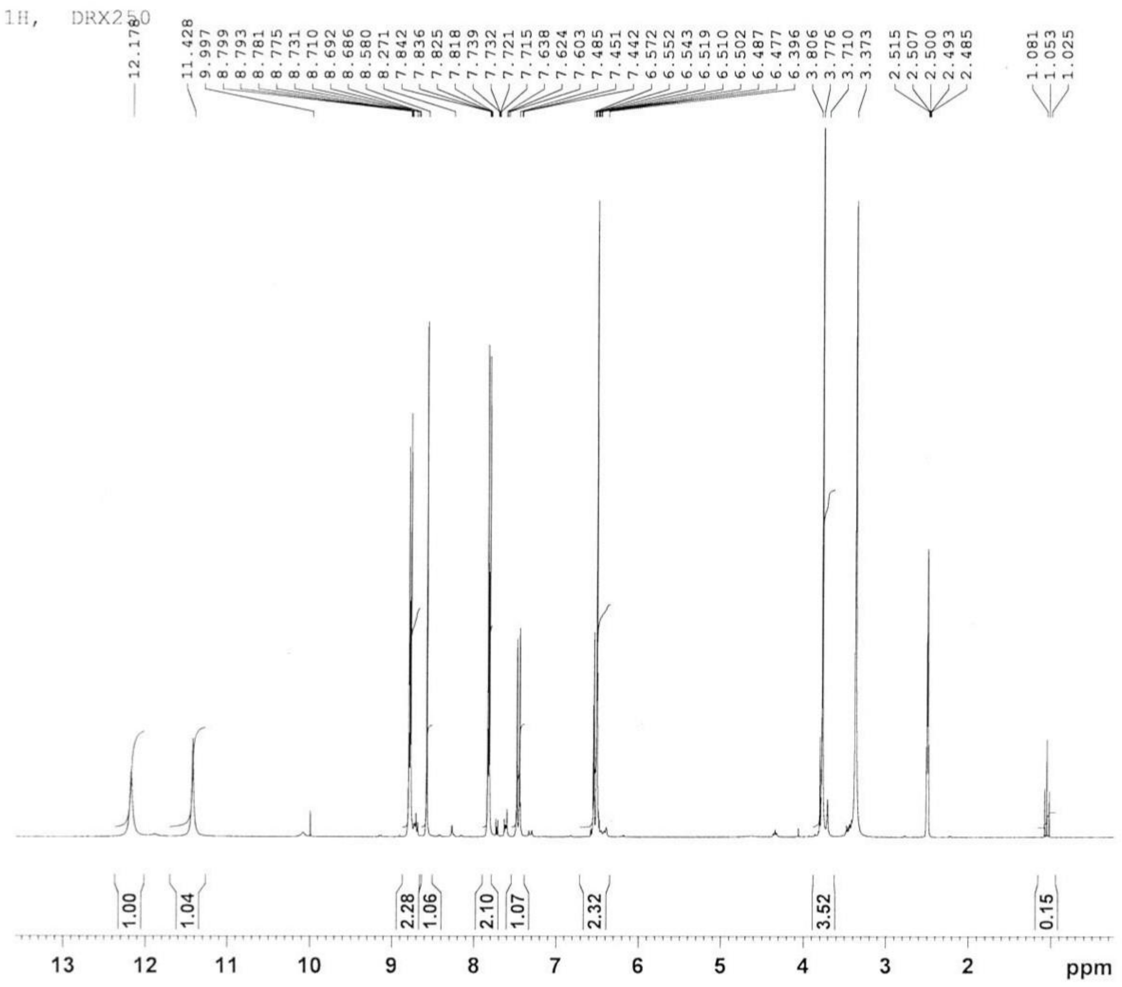

Figure S8. ^{1}H NMR spectra of the compound **14**

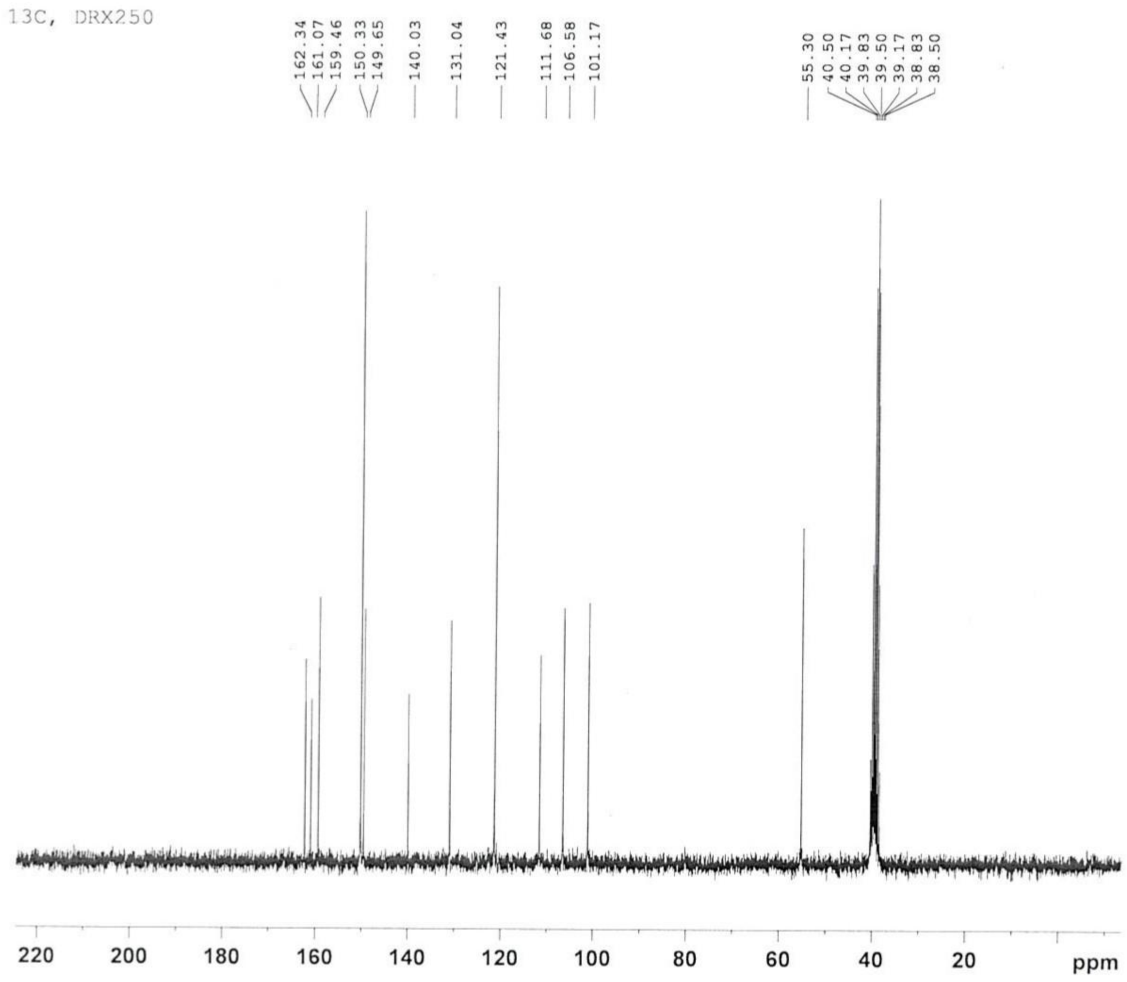

Figure S9. ^{13}C NMR spectra of the compound **14**
